# Association between *in vitro* fertilization and preeclampsia: a multicenter cohort study with subgroup analysis by maternal age, BMI, and parity

**DOI:** 10.3389/fendo.2026.1755506

**Published:** 2026-05-15

**Authors:** Ping Yu, Sha Chen, Qiong Li, Jingyang Li, Deshen Han, Ying Gu, Yu Chen, Chaoyan Yue

**Affiliations:** 1 Center of Reproductive Medicine, Women’s Hospital of Jiangnan University, Wuxi Maternity and Child Health Care Hospital, Wuxi, China; 2 Nanjing Medical University, Wuxi Medical Center, Wuxi, China; 3 Department of Obstetrics and Gynecology, The First People's Hospital of Chenzhou, Hengyang Medical School, University of South China, Chenzhou, China; 4 Department of Obstetrics, Affiliated Women's Hospital of Jiangnan University, Wuxi Maternity and ChildHealth Care Hospital, Wuxi, Jiangsu, China; 5 Wuxi School of Medicine, Jiangnan University, Wuxi, China; 6 Department of Obstetrics, Wuxi Maternal and Child Health Care Hospital, Wuxi Medical Center, Nanjing Medical University, Wuxi, China; 7 Obstetrics & Gynecology Hospital of Fudan University, Shanghai Key Lab of Reproduction and Development, Shanghai Key Lab of Female Reproductive Endocrine Related Diseases, Shanghai, China

**Keywords:** *in vitro* fertilization, multicenter cohort study, preeclampsia, preterm birth, small for gestational age

## Abstract

**Background:**

Preeclampsia (PE) is a significant complication of pregnancy, and its association with *in vitro* fertilization (IVF) remains unclear, especially across different demographic groups.

**Methods:**

We conducted a retrospective multicenter cohort study including 61,329 singleton pregnancies from three hospitals in China, with 3,902 conceived via IVF and 57,427 conceived naturally. Multivariate logistic regression and Kaplan-Meier analyzes were used to assess the associations between IVF and preeclampsia, preterm birth, and small for gestational age (SGA), adjusting for maternal age, BMI, parity, and other clinical variables. Subgroup analyzes were performed by age (< 35 *vs*. ≥ 35), BMI (< 24 *vs*. ≥ 24 kg/m²), and parity.

**Results:**

IVF was significantly associated with increased risks of preeclampsia (adjusted OR = 1.34, 95% CI: 1.14–1.57, *p =* 0.0004) and preterm birth (adjusted OR = 1.68, 95% CI: 1.43–1.97, *p* < 0.0001), but not with SGA. Subgroup analyzes showed that the association between IVF and preeclampsia was consistent across subgroups defined by age, BMI, and parity, with no significant interactions. For preterm birth, a significant age interaction was observed (*p =* 0.0005), with a stronger association in women under 35 years.

**Conclusion:**

IVF is independently associated with an increased risk of preeclampsia and preterm birth. Enhanced monitoring and tailored prenatal care are warranted in IVF pregnancies.

## Introduction

Assisted reproductive technology (ART) is vital for offering hope to infertile patients seeking successful pregnancies. Between ten million and 13 million or more infants have been born through ART in the 40 years since the first such birth in 1978. This substantial number of infants, resulting from both conventional and innovative ART applications, confirms that ART has enabled millions to achieve parenthood, is now a mainstream medical practice, and has had a significant societal impact (1). ART is widely practiced globally but still exhibits significant disparities in utilization, practice, effectiveness, and safety (2). There is a recognized association between ART and adverse perinatal outcomes, which include both short-term and long-term health risks (3). Preeclampsia, a significant complication specific to pregnancy, remains inadequately understood regarding its epidemiological traits and underlying mechanisms in patients undergoing IVF. Additionally, current research has revealed considerable geographical and racial variations in the incidence of Preeclampsia (4).

The risks associated with ART technology and their long-term effects are crucial to national population quality. While earlier research has shown that women undergoing IVF have a significantly greater likelihood of developing preeclampsia compared to those experiencing natural pregnancies, alongside a heightened occurrence of preterm births and infants classified as SGA (5–7), a number of elements related to these connections continue to be quite contentious. Various theoretical frameworks, including placental dysfunction, dysregulation of the immune system, and injury to vascular endothelium, have been suggested to clarify the association between IVF and preeclampsia (8, 9). The connection between IVF and SGA is still a matter of vigorous discussion (5, 10).

Maternal age, BMI, and parity each influence preeclampsia risk in pregnancy. But whether they change how IVF relates to preeclampsia is less clear. Some studies indicate that older women undergoing IVF have higher preterm birth rates, while others do not (11–14). And we still don’t know how age specifically affects the IVF-PE link. For BMI, obesity clearly raises preeclampsia risk in IVF patients (15), but we need Chinese data to see if the pattern holds across different BMI cutoffs. Parity is associated with a reduced risk of adverse outcomes such as preterm birth and SGA seem less common in women who have already given birth (10), suggesting first-time mothers may be more vulnerable to IVF-related risks. The problem is, most previous studies were too small, used single centers, or lacked the statistical power to test these interactions properly, especially in Chinese populations.

We designed this study using a large, multicenter cohort from three Chinese hospitals. Our primary aim was to determine if IVF independently predicts preeclampsia, preterm birth, and SGA after adjusting for age, BMI, parity, and other clinical factors, and whether IVF-associated risks differ by age, BMI, or parity. With our sample size and multicenter design, we have sufficient statistical power to test these interactions. We believe this will help clinicians better tailor risk assessment and prenatal care for women undergoing IVF.

## Methods

### Patients and design

This study used a retrospective cohort design conducted from January 2018 to June 2024, utilizing data collected from three medical facilities: the Obstetrics and Gynecology Hospital of Fudan University (located in Shanghai, China; n = 39,328), Wuxi Maternal and Child Health Hospital (Wuxi, China; n = 16,159), and Chenzhou First People’s Hospital (Chenzhou, China; n = 5,842). Multiple gestations were excluded during data extraction, so only singleton pregnancies were included in the analysis. Detailed clinical and anthropometric information was gathered from 3,902 women who underwent *in vitro* fertilization (referred to as the IVF group) and 57,427 women who achieved spontaneous conception (designated as the NC group). Each participant was assessed for preeclampsia and provided comprehensive medical histories. To qualify for inclusion, complete data for all essential variables was required. Individuals with chronic hypertension, multiple births, and those without complete maternal or neonatal records were excluded from the study. Data on maternal and infant outcomes were obtained from the Hospital Information System (HIS) and the Laboratory Information Management System (LIS). This research adhered to the ethical principles outlined in the Declaration of Helsinki (1964 and its subsequent amendments) and received approval from the ethics review boards of the involved institutions.

### Variables and measurements

The exposure of interest was the use of IVF. Initial laboratory assessments were performed during the first clinical encounter. The analysis accounted for potential confounders, including age, parity, BMI, systolic and diastolic blood pressure, aspirin usage, antihypertensive therapy, family history of diabetes and hypertension, as well as serum levels of ALT and creatinine. These measurements were obtained at the first prenatal visit, which occurred prior to the diagnostic time of preeclampsia (after 20 weeks of gestation), thereby reducing the risk of reverse causality.

### Outcomes

The primary outcome was preeclampsia, diagnosed as the onset of hypertension in a woman with previously normal blood pressure after 20 weeks of gestation, accompanied by proteinuria or other end-organ dysfunction. according to the American College of Obstetricians and Gynecologists (ACOG) guidelines (16). Postpartum preeclampsia was not included in this study. The secondary outcomes comprised preterm birth (defined as delivery prior to 37 weeks of gestation (17)) and the occurrence of SGA(defined as a birthweight that falls below the 10th percentile of the distribution of newborns’ birthweights at the same gestational age according to a global reference (18).). Gestational age was assessed using ultrasound measurements conducted during the first trimester, and all necessary data were obtained from the HIS records.

### Statistical analysis

Continuous variables are expressed as mean ± standard deviation (SD), while categorical variables are reported as frequencies (%). Odds ratios (ORs) were obtained through multivariate logistic regression models that incorporated adjustments for potential confounders to account for potential confounders of the findings. The analysis was stratified by maternal age, BMI, and parity. Furthermore, sensitivity analyzes were performed within subgroups to assess the stability of the relationship between IVF and the outcomes of preeclampsia and preterm birth. Interaction tests were conducted to explore variations in ORs across different subgroups. Although preterm birth is a binary outcome, we utilized Kaplan-Meier survival analysis to visualize the cumulative incidence of preterm birth over gestational age. Gestational age was used as the time scale, with preterm birth (<37 weeks of gestation) defined as the event. Pregnancies that reached 37 weeks or beyond without preterm birth were considered to have exited the risk set at 37 weeks, marking the end of the preterm birth risk window. Accordingly, the Kaplan–Meier curve was used to illustrate the temporal distribution of preterm birth across gestation by conception mode. All statistical analyzes were carried out using IBM SPSS (version 21.0; IBM, Armonk, NY) and R software (version 3.4.3; The R Foundation; https://www.r-project.org). A two-tailed P-value < 0.05 was considered statistically significant. Interaction tests were conducted to assess subgroup heterogeneity in preterm birth risk between IVF and naturally conceived pregnancies across the age strata.

## Result

### Comparison of baseline characteristics

The study included 57,427 participants in the NC group and 3,902 in the IVF group. **Table 1** summarizes all baseline characteristics. The total number for some outcomes is smaller than the overall study population due to outcome-specific missing data; subsequent analyzes were based on the available data for each outcome. Participants in the IVF group were significantly older and had a slightly higher mean BMI than those in the NC group. Systolic and diastolic blood pressure levels were also significantly elevated in the IVF group. Liver function, as indicated by ALT levels, showed a small but significant increase in the IVF group. Medication use, including aspirin, was more common in the IVF group, and a higher prevalence of family history of diabetes was noted in this group.

**Table 1:** Baseline characteristics.

Characteristic	NC n= 57427	IVF n= 3902	*p*
Preeclampsia			< 0.001
No	54754 (95.35%)	3505 (89.83%)	
Yes	2673 (4.65%)	397 (10.17%)	
Preterm Birth			< 0.001
No	40866 (91.99%)	2392 (81.25%)	
Yes	3557 (8.01%)	552 (18.75%)	
SGA			< 0.001
No	55071 (95.93%)	3678 (94.28%)	
Yes	2338 (4.07%)	223 (5.72%)	
AGE	31.20 ± 4.15	33.52 ± 4.04	< 0.001
BMI	21.80 ± 3.21	22.22 ± 3.20	< 0.001
Systolic pressure	115.45 ± 12.43	118.07 ± 12.56	< 0.001
Diastolic pressure	69.56 ± 9.47	72.99 ± 9.78	< 0.001
ALT	18.21 ± 16.13	19.23 ± 17.44	0.002
Creatinine	40.65 ± 10.40	40.91 ± 10.77	0.191
Parity			< 0.001
No	38770 (68.65%)	3153 (82.93%)	
Yes	17702 (31.35%)	649 (17.07%)	
Aspirin			< 0.001
No	56704 (98.74%)	3727 (95.52%)	
Yes	723 (1.26%)	175 (4.48%)	
Depressor			< 0.001
No	57062 (99.36%)	3847 (98.59%)	
Yes	365 (0.64%)	55 (1.41%)	
Family history of hypertension			0.205
No	49836 (86.80%)	3359 (86.08%)	
Yes	7582 (13.20%)	543 (13.92%)	
Family history of diabetes			0.009
No	54275 (94.53%)	3650 (93.54%)	
Yes	3143 (5.47%)	252 (6.46%)	

IVF, *in vitro* fertilization; NC, natural conception; SGA, small for gestational age; BMI, body mass index; ALT, alanine aminotransferase.

The rate of preeclampsia was more than twice as high in the IVF group (10.17% vs. 4.65%, *p* < 0.001). Additionally, the IVF group had a higher proportion of nulliparous women. Pregnancy outcomes also differed significantly, with higher rates of preterm birth (18.75% *vs*. 8.01%, *p* < 0.001) and a higher incidence of SGA infants (5.72% *vs*. 4.07%, *p* < 0.001) in the IVF group.

### Univariate analysis

In unadjusted comparisons, IVF was associated with higher odds of preeclampsia (OR = 2.32, 95% CI: 2.08–2.59, *p* < 0.001), preterm birth (OR = 2.65, 95% CI: 2.40–2.93, *p* < 0.001), and SGA (OR = 1.43, 95% CI: 1.24–1.65, *p* < 0.001) compared to natural conception (**Table 2**).

**Table 2:** Odds ratio of primary and secondary outcomes.

Exposure	Odds ratio (95% CI)	*p*	Adjust odds ratio (95% CI)	*p*
Preeclampsia				
IVF				
No	Reference		Reference	
Yes	2.32 (2.08, 2.59)	< 0.0001	1.34 (1.14, 1.57)	0.0004
Preterm Birth				
IVF				
No	Reference		Reference	
Yes	2.65 (2.40, 2.93)	< 0.0001	1.68 (1.43, 1.97)	< 0.0001
SGA				
IVF				
No	Reference		Reference	
Yes	1.43 (1.24, 1.65)	< 0.0001	1.16 (0.97, 1.37)	0.0985

Adjusted for age, parity, BMI, Systolic pressure, Diastolic pressure, Aspirin Depressor, family history of hypertension, family history of diabetes, ALT and CR.

IVF, *in vitro* fertilization; SGA, small for gestational age; BMI, body mass index; ALT, alanine aminotransferase; CR, creatinine.

### Multivariate regression analysis

After adjusting for age, parity, BMI, systolic pressure, diastolic pressure, aspirin use, depressor use, family history of hypertension, family history of diabetes, ALT levels, and CR levels, IVF remained independently associated with preeclampsia (adjusted OR = 1.34, 95% CI: 1.14–1.57, *p =* 0.0004) and preterm birth (adjusted OR = 1.68, 95% CI: 1.43–1.97, *p* < 0.0001). The association between IVF and SGA, however, was no longer significant after adjustment (adjusted OR = 1.16, 95% CI: 0.97–1.37, *p =* 0.0985) (**Table 2**). The univariate difference in SGA incidence in the IVF group disappeared after adjusting for confounding factors, suggesting that this association may be mediated by factors such as age and BMI rather than being an independent effect of IVF.

### Subgroup analysis

**Table 3** shows the association between IVF and preeclampsia across subgroups of age, BMI, and parity. After adjusting for confounders, IVF was linked to a higher risk of preeclampsia in women under 35 (adjusted OR = 1.45, 95% CI: 1.19–1.76, *p =* 0.0002), but not in those aged 35 or older (adjusted OR = 1.14, 95% CI: 0.86–1.51, *p =* 0.3533). The association was significant in both BMI groups: below 24 kg/m² (adjusted OR = 1.33, 95% CI: 1.09–1.61, *p =* 0.0047) and 24 kg/m² or higher (adjusted OR = 1.36, 95% CI: 1.03–1.79, *p =* 0.0292), as well as in both parity groups (primiparous: adjusted OR = 1.32, 95% CI: 1.11–1.56, *p =* 0.0014; multiparous: adjusted OR = 1.58, 95% CI: 1.01–2.49, *p =* 0.0460). Interaction tests revealed no significant effect modification by age (*p =* 0.3858), BMI (*p =* 0.864), or parity (*p =* 0.2894), suggesting that the IVF-PE association remains stable across subgroups with different clinical characteristics.

**Table 3:** Subgroup analysis of preeclampsia and IVF.

Exposure	Non-adjusted model (95% CI)	*p*	*p* value for interaction	Adjust model (95% CI)	*p*	*p* value for interaction
Age			0.1479			0.3858
<35						
IVF						
No	Reference			Reference		
Yes	2.35 (2.05, 2.70)	< 0.0001		1.45 (1.19, 1.76)	0.0002	
≥35						
IVF						
No	Reference			Reference		
Yes	1.98 (1.64, 2.39)	< 0.0001		1.14 (0.86, 1.51)	0.3533	
BMI			0.0171			0.864
<24						
IVF						
No	Reference			Reference		
Yes	2.56 (2.22, 2.95)	< 0.0001		1.33 (1.09, 1.61)	0.0047	
≥24						
IVF						
No	Reference			Reference		
Yes	1.93 (1.60, 2.32)	< 0.0001		1.36 (1.03, 1.79)	0.0292	
Parity			0.615			0.2894
Primipara						
IVF						
No	Reference			Reference		
Yes	2.15 (1.91, 2.43)	< 0.0001		1.32 (1.11, 1.56)	0.0014	
Multipara						
IVF						
No	Reference			Reference		
Yes	2.34 (1.73, 3.17)	< 0.0001		1.58 (1.01, 2.49)	0.0460	

Adjusted for age, parity, BMI, Systolic pressure, Diastolic pressure, Aspirin Depressor, family history of hypertension, family history of diabetes, ALT and CR.

IVF, *in vitro* fertilization; BMI, body mass index; ALT, alanine aminotransferase; CR, creatinine.

**Table 4** presents the association between IVF and preterm birth across subgroups of age, BMI, and parity. After adjusting for confounders, IVF was associated with a higher risk of preterm birth in women under 35 years of age (adjusted OR = 2.01, 95% CI: 1.66–2.42, *p* < 0.0001), in both BMI groups (< 24 kg/m²: adjusted OR = 1.50, 95% CI: 1.24–1.82, *p* < 0.0001; ≥ 24 kg/m²: adjusted OR = 2.09, 95% CI: 1.55–2.83, *p* < 0.0001), and in primiparous women (adjusted OR = 1.67, 95% CI: 1.40–2.00, *p* < 0.0001). However, this association was not observed in women aged 35 or older (adjusted OR = 1.03, 95% CI: 0.75–1.41, *p =* 0.8506) or in multiparous women (adjusted OR = 1.38, 95% CI: 0.92–2.07, *p =* 0.1216). Interaction tests indicated that only age significantly modified the relationship between IVF and preterm birth (*p =* 0.0005), while no significant interactions were found for BMI (*p =* 0.106) or parity (*p =* 0.346). These findings suggest that, aside from age, the association between IVF and preterm birth remains consistent across different BMI and parity subgroups.

**Table 4:** Subgroup analysis of preterm birth and IVF.

Exposure.	Non-adjusted model (95% CI)	*p*	*p* value for interaction	Adjust model (95% CI)	*p*	*p* value for interaction
Age			< 0.0001			0.0005
<35						
IVF						
No	Reference			Reference		
Yes	3.07 (2.73, 3.46)	< 0.0001		2.01 (1.66, 2.42)	< 0.0001	
≥35						
IVF						
No	Reference			Reference		
Yes	1.68 (1.40, 2.01)	< 0.0001		1.03 (0.75, 1.41)	0.8506	
BMI			0.0115			0.106
<24						
IVF						
No	Reference			Reference		
Yes	2.46 (2.17, 2.79)	< 0.0001		1.50 (1.24, 1.82)	< 0.0001	
≥24						
IVF						
No	Reference			Reference		
Yes	3.26 (2.73, 3.89)	< 0.0001		2.09 (1.55, 2.83)	< 0.0001	
Parity			0.0032			0.3458
Primipara						
IVF						
No	Reference			Reference		
Yes	2.92 (2.62, 3.27)	< 0.0001		1.67 (1.40, 2.00)	< 0.0001	
Multipara						
IVF						
No	Reference			Reference		
Yes	1.98 (1.55, 2.52)	< 0.0001		1.38 (0.92, 2.07)	0.1216	

Adjusted for age, parity, BMI, Systolic pressure, Diastolic pressure, Aspirin Depressor, family history of hypertension, family history of diabetes, ALT, CR and preeclampsia.

IVF, *in vitro* fertilization; BMI, body mass index; ALT, alanine aminotransferase; CR, creatinine.

### Kaplan-Meier analysis

**Figure 1:**
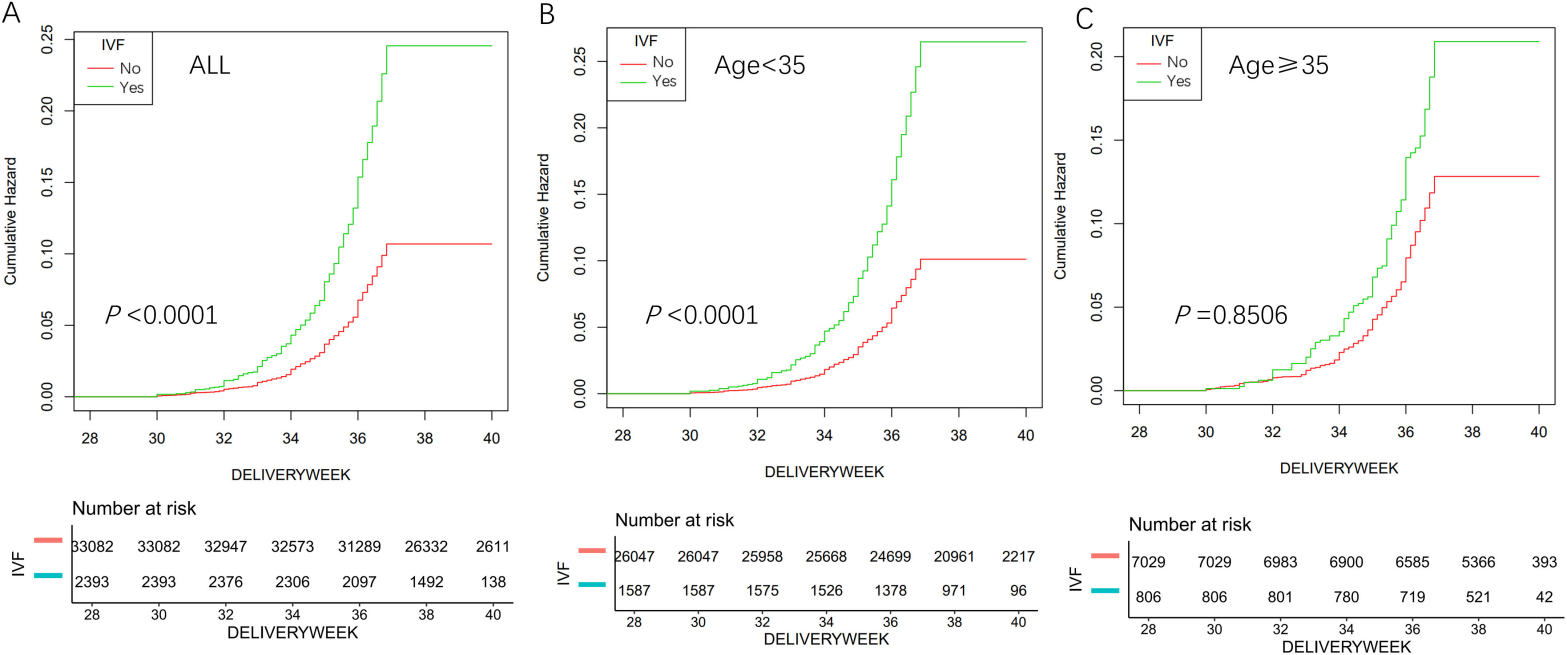
Cumulative risk of preterm birth at different ages in the cohort. **(A)** Overall cumulative risk of preterm birth. **(B)** Cumulative risk of preterm birth at age < 35 years. **(C)** Cumulative risk of preterm birth at age ≥ 35 years.

**Figure 1** shows the cumulative risk of preterm birth over gestational weeks, broken down by maternal age. A significantly higher cumulative risk of preterm birth was observed in the IVF group compared with the naturally conceived (NC) group (*p* < 0.0001; **Figure 1A**). A statistically significant interaction between IVF exposure and maternal age was identified (*p* for interaction= 0.0005 in adjusted models; **Figure 1B, C**). Women aged <35 years undergoing IVF exhibited a markedly elevated cumulative risk of preterm birth (*p* < 0.001; **Figure 1B**), consistent with the stratified odds ratios reported in **Table 4**. In contrast, no significant association was observed among women aged ≥35 years (*p =* 0.851; **Figure 1C**).

## Discussion

### Main results

After adjusting for maternal age, BMI, parity, blood pressure, medication use, and other clinical covariates, IVF was associated with a 34% higher risk of preeclampsia (adjusted OR = 1.34, 95% CI: 1.14–1.57, *p =* 0.0004) and a 68% higher risk of preterm birth (adjusted OR = 1.68, 95% CI: 1.43–1.97, *p* < 0.0001) compared with natural conception. No clear association was found between IVF and SGA after adjustment (adjusted OR = 1.16, 95% CI: 0.97–1.37, *p =* 0.0985).

Subgroup analyzes showed the IVF-preeclampsia link was consistent across age, BMI, and parity subgroups, with no significant interactions (age: *p =* 0.3858; BMI: *p =* 0.864; parity: *p =* 0.2894). For preterm birth, the association also held across BMI and parity subgroups (BMI: *p =* 0.106; parity: *p =* 0.346), but age modified the IVF-preterm birth relationship (p for interaction = 0.0005). The association was stronger in women under 35 (adjusted OR = 2.01, 95% CI: 1.66–2.42) than in those aged 35 or older (adjusted OR = 1.03, 95% CI: 0.75–1.41). Kaplan-Meier analysis confirmed higher cumulative preterm birth risk in IVF pregnancies, especially among younger women (*p* < 0.0001).

### Consistency and disagreement with previous evidence

Our research revealed a significant increase in the risk of preeclampsia among pregnant women who undergo IVF, which aligns with findings from China (aRR = 1.54; 95% CI: 1.51–1.57) and meta-analyzes conducted in Western populations (RR = 1.71; 95% CI: 1.11–2.62) (6, 19). This suggests that the elevated risk of preeclampsia associated with IVF has been consistently observed across different populations. The same goes for preterm birth. One study found an OR of 2.06 (95% CI: 1.16–3.68) (5). Another study demonstrated that IVF was significantly associated with elevated preterm birth risk (OR = 1.95, 95% CI: 1.76-2.15) (20). These data substantiate the statistical correlation between IVF and preterm birth. Our finding (OR = 1.68, 95% CI: 1.43-1.97) aligns with previous investigations, demonstrating consistency with earlier reported associations. Conversely, in our study, no statistically significant link was found between IVF pregnancies and SGA, which contrasts with results from some research (5, 10). While current literature indicates a connection between IVF and higher risks of both Preterm birth and SGA, these risks seem to diminish with increased parity (10). This suggests that the higher SGA rate observed in IVF pregnancies may be explained by other factors, such as parity, rather than IVF itself.

Our results differ from a study by Li et al. (2025), who reported that IVF increased the risk of preeclampsia specifically in women aged 35 or older (aRR = 1.52) using single-center data from Shanghai (21). Several factors may account for this difference. The two studies adjusted for different confounders: we included blood pressure, medication use, and laboratory values, whereas Li et al. adjusted for a broader range of reproductive conditions. Additionally, our data came from three hospitals across distinct regions of China, compared with their single-center data. Differences in baseline characteristics or statistical power may also have contributed.

For BMI, we found that IVF increased the risk of preeclampsia in both leaner and heavier women, with similar odds ratios (ORs) (1.33 vs. 1.36). This finding aligns with Liu et al. (2020), who reported that pregravid obesity independently increased preeclampsia risk in IVF pregnancies (OR = 2.92) (15). Our data extend this finding by showing that the association holds across a wider BMI range, not only in obese women. The similar ORs across BMI strata suggest that IVF adds a consistent relative risk regardless of body size.

The relationship between maternal age and preterm birth risk in women undergoing IVF remains a topic of debate in the existing literature. Some studies found no significant age association (12, 13)., while others reported increased risk with advanced age (11, 14). Our research highlights that IVF increased the risk of preterm birth in women under 35 (adjusted OR = 2.01, 95% CI: 1.66–2.42) but had no detectable effect in those 35 or older (adjusted OR = 1.03, 95% CI: 0.75–1.41). The interaction test confirmed that age significantly modifies the IVF-preterm birth relationship (*p* for interaction = 0.0005).This pattern suggests that in younger women who are otherwise at low risk, the IVF procedure itself may contribute more to preterm birth. In older women, age-related factors may dominate, leaving less room for IVF to show an independent effect.

### Mechanistic considerations

IVF may increase the risk of preeclampsia through several biological pathways.

#### Estrogen and placentation

Controlled ovarian stimulation increases estradiol levels during IVF cycles. Martin et al. (2016) discovered that estradiol levels above the 90th percentile raised the risk of preeclampsia from 4.5% to 18.5% (22). A baboon study demonstrated that high estrogen levels can reduce trophoblast invasion of spiral arteries (23). When this invasion is impaired, it leads to inadequate spiral artery remodeling, which decreases placental blood flow—a well-established pathway to preeclampsia (24)These estrogen effects on placentation are common to all IVF cycles, which helps explain the consistent IVF-PE association observed across subgroups in our study.

#### Placental adaptation and parity

Burke et al. (2023) examined placental tissue from IVF pregnancies. Primiparous women were more likely to exhibit maternal vascular malperfusion (aOR = 0.6 for multiparity vs. primiparity), a sign of inadequate spiral artery remodeling. In contrast, multiparous women were more likely to show delayed villous maturation (aOR = 4.9) (25). Thus, parity influences placental histopathological features. Across both parity groups, IVF remained consistently associated with an increased risk of preeclampsia.

#### BMI and baseline risk

The odds ratios for IVF-related preeclampsia (IVF-PE) were quite similar across different BMI categories (1.33 vs. 1.36), suggesting that IVF consistently adds to the relative risk of preeclampsia, regardless of a person’s body size. It seems that IVF may increase preeclampsia risk through pathways that are independent of BMI, with the primary mechanism being supraphysiological estrogen-induced abnormal placentation. This mechanism appears to work similarly across all BMI groups. Kluge et al. (2023) reported that the absolute risk of preeclampsia increases with higher BMI, ranging from 4.6% in women with a normal weight to 20.3% in those with class III obesity (26). This reflects a higher baseline risk in obese women due to chronic inflammation, but this baseline difference does not change the relative effect of IVF. Similarly, Dayan et al. (2018) found no statistical interaction between high BMI and IVF regarding severe maternal complications, indicating that these two factors likely act through independent pathways (27).

#### Age and preterm birth

IVF increased preterm birth risk in women under 35 (OR = 2.01) but not in those aged 35 or older (OR = 1.03). The mechanism underlying this age-related difference remains unclear. One possibility is that younger women have stronger ovarian responses to controlled stimulation, leading to higher estrogen levels and an altered endometrial environment that may trigger early labor. Li et al. (2022) similarly reported the highest preterm birth risk among IVF/ICSI patients in women under 25 (OR = 2.13) (11). In older women, age-related factors such as cervical insufficiency, uterine fibroids, or vascular changes may predominate, leaving less room for IVF to exert an independent effect. Whether high estrogen directly contributes to preterm birth remains uncertain and warrants further investigation.

### Clinical significance

Our findings, which indicate that IVF is independently linked to an increased risk of PE and preterm birth, hold significant clinical importance. Regarding preeclampsia, the association between IVF and this condition remained consistent across subgroups defined by age, BMI, and parity, with no notable interactions observed. As such, all women undergoing IVF should receive routine preeclampsia surveillance in accordance with standard clinical guidelines. Different IVF procedures may also influence pregnancy outcomes. For example, a comparative study by Lian et al. in patients with diminished ovarian reserve showed that while the Luteal-Phase Ovarian Stimulation (LPOS) protocol was independently linked to a higher mature oocyte yield, the Progestin-Primed Ovarian Stimulation (PPOS) protocol required a significantly shorter stimulation duration and lower total gonadotropin dose, suggesting that individualized stimulation strategies could help optimize outcomes (28). Additionally, since low-dose aspirin is currently recognized as an effective preventive measure for preeclampsia in high-risk populations (29), our results support considering IVF as a predictive factor for preeclampsia. This insight may enable future disease prevention through targeted aspirin prophylaxis in appropriately selected patients. Future research should integrate IVF procedural details and longitudinal biomarker assessments to further refine these prevention strategies.

### Advantages and limitations

The advantages of this study are the multi-center design and the strict correction of confounding factors. However, limitations exist. The retrospective design prevents us from inferring causality, and residual confounding from unmeasured variables is possible. We lacked details on IVF protocols (such as fresh versus frozen transfer and stimulation type) because reproductive and obstetric records are maintained separately at our centers. These factors may modify the risk of preeclampsia but could not be adjusted for. Future prospective studies should systematically collect these variables to better isolate the independent effect of IVF. Furthermore, we had no data on infertility etiology (e.g., polycystic ovary syndrome, endometriosis, tubal factor), smoking status, or socioeconomic indicators, as these were not consistently recorded across centers. Future studies should link reproductive and obstetric databases and collect comprehensive data on these factors to better understand how IVF procedures affect pregnancy outcomes. We did not differentiate preeclampsia by severity or by timing of onset. The pathophysiology and IVF association may differ across these subtypes. Future studies with larger sample sizes should stratify preeclampsia by severity and gestational age at onset.

## Conclusion

The study demonstrates that women undergoing IVF have a higher likelihood of developing preeclampsia. Subgroup analyzes showed that the association between IVF and preeclampsia was consistent across subgroups defined by age, BMI, and parity, with no significant interactions. For preterm birth, a significant age interaction was observed, with a stronger association in women under 35. Although IVF is associated with an increased risk of certain complications, these risks can be mitigated through individualized clinical management, including optimized ovulation stimulation protocols and enhanced prenatal monitoring. Future research should integrate detailed IVF procedural data and longitudinal biomarkers to further refine risk prediction and elucidate potential mechanisms.

## Data Availability

The original contributions presented in the study are included in the article/supplementary material. Further inquiries can be directed to the corresponding authors.
